# Interplay between HIV-1 and Host Genetic Variation: A Snapshot into Its Impact on AIDS and Therapy Response

**DOI:** 10.1155/2012/508967

**Published:** 2012-05-16

**Authors:** Raghavan Sampathkumar, Elnaz Shadabi, Ma Luo

**Affiliations:** ^1^Department of Medical Microbiology, University of Manitoba, Winnipeg, MB, Canada R3E 0J9; ^2^National Microbiology Laboratory, Public Health Agency of Canada, Winnipeg, MB, Canada R3E 3R2

## Abstract

As of February 2012, 50 circulating recombinant forms (CRFs) have been reported for HIV-1 while one CRF for HIV-2. Also according to HIV sequence compendium 2011, the HIV sequence database is replete with 414,398 sequences. The fact that there are CRFs, which are an amalgamation of sequences derived from six or more subtypes (CRF27_cpx (cpx refers to complex) is a mosaic with sequences from 6 different subtypes besides an unclassified fragment), serves as a testimony to the continual divergent evolution of the virus with its approximate 1% per year rate of evolution, and this phenomena *per se* poses tremendous challenge for vaccine development against HIV/AIDS, a devastating disease that has killed 1.8 million patients in 2010. Here, we explore the interaction between HIV-1 and host genetic variation in the context of HIV/AIDS and antiretroviral therapy response.

## 1. Introduction

The evidence for HIV to be the causative agent of AIDS was documented way back in 1983, and, hitherto, the dreadful HIV remains unconquered [1]. As of 2010, 34 million people are living with HIV infections and 2.7 million people have been newly infected in that year alone [2]. This alarming statistics have accelerated much research into the biology of HIV, seeking clues on “Achilles heel” so as to curtail its spread and eventually to eradicate it.

## 2. HIV-1 Origin and Diversity

HIV-1 and HIV-2 cause AIDS, and HIV-1, with its tremendous diversity, outwits HIV-2 by its ability to inflict a more virulent form of the disease and has global distribution [3]. Both viruses originated in Africa, and viral zoonosis resulted in the rampant AIDS epidemic. Simian immunodeficiency virus (SIV) from chimpanzees (SIV_CPZ_) is closely related to HIV-1, while SIV from sooty mangabeys (SIV_SM_) forms the closest to HIV-2 [4, 5]. HIV-1 viruses fall under three main phylogenetic lineages, namely, M (Main), O (outlier), and N (non-M/non-O), all considered to have originated from chimpanzees dwelling in the eastern equatorial forests of Cameroon, West Central Africa, with O group viruses through a gorilla intermediate [6–8]. SIV infected *Pan troglodytes troglodytes* (Ptt) chimpanzees gave rise, through cross-species transmission, to HIV-1 groups M and N viruses while SIV-infected gorillas (*Gorilla gorilla*; SIVgor), which themselves contracted infection originally from chimpanzees, gave rise to formation of group O HIV-1 viruses. Recently, a variant of HIV-1 group O virus has been detected—P group—which resembles more closely to SIVgor than O group virus, in individuals of Cameroon origin [9, 10]. Studies have estimated the timing for origin of each lineage of HIV—HIV-1 group M, O, and N at 1931 (1915–1941), 1920 (1890–1940), and 1963 (1948–1977), respectively [11–13]. HIV-2 viruses are considered to have originated around 1930s [13]. 

**Figure 1:**
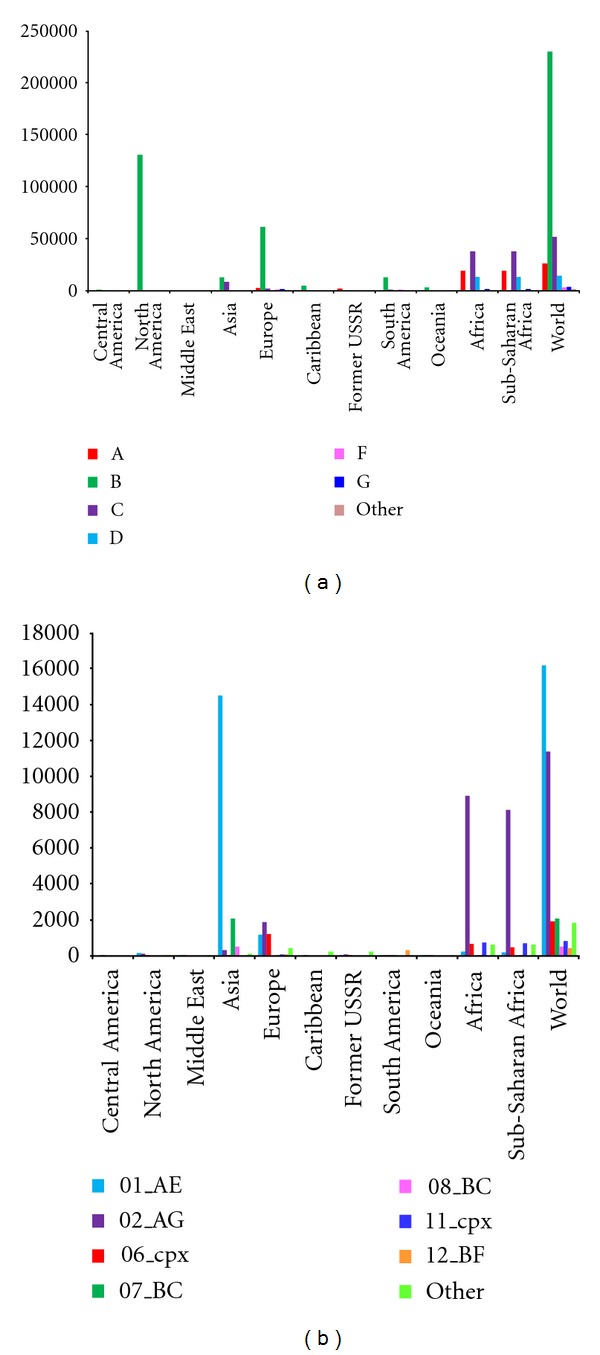
(a) Global distribution of HIV-1 (nonrecombinants) sequences. (b) Global distribution of recombinant HIV-1 sequences. Source: HIV databases [14].

**Figure 2:**
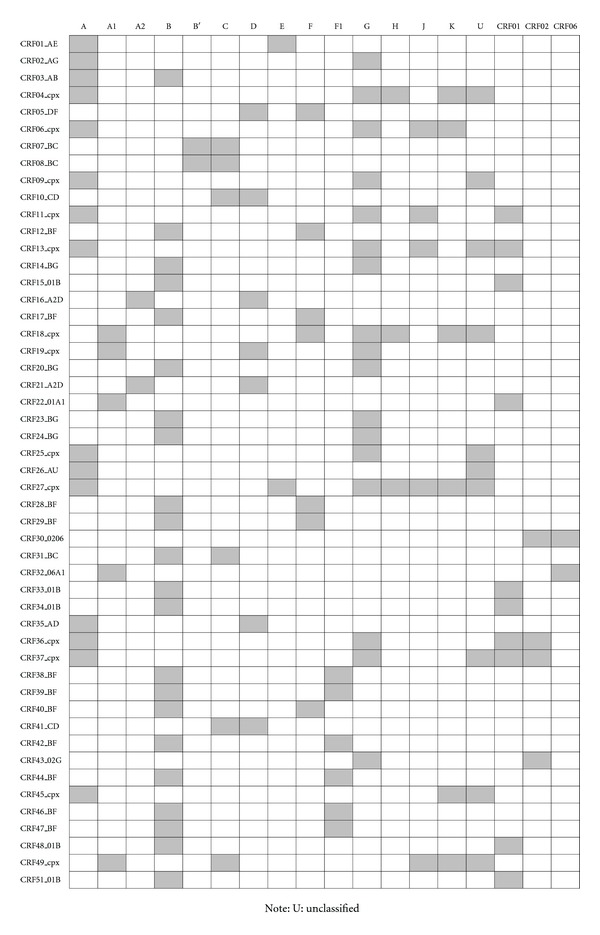
Schematic representation of composition of HIV-1 CRFs.

Group M HIV-1 viruses are further subdivided into nine major subtypes, namely, A-D, F-H, J, and K. Sub-subtypes have been reported for clade A (A1 and A2) and F (F1 and F2) viruses. Group M also includes circulating recombinant forms (CRFs). Figures 1(a) and 1(b) illustrate global distribution of HIV subtypes and most common CRFs, respectively. Circulating recombinant forms arise as a result of recombination between any of the subtypes and/or CRFs leading to the formation of CRFs such as AE, AG, AB, DF, BC, CD, and other complex forms.  Figure 2 provides a schematic representation of composition of 50 CRFs that have been identified hitherto [14]. The subtypes and CRFs attest to the genetic diversity of HIV-1. These M group viruses cause most of the HIV-1 infections, accounting for the current AIDS pandemic. Circulating recombinant forms account for about 10% of all HIV infections [8], and the proportion might increase in future. Different subtypes among HIV-1 viruses impact disease progression differently [15] and may also have differential sensitivity to antiretroviral therapy (ART) drugs [16]. Individuals infected with subtype D viruses are known to experience rapid disease progression [17], whereas those infected with subtype C undergo slow disease progression [18]. During the course of HIV-1 infection, strains that utilize coreceptor CXCR4, emerge during late stages of infection in contrast to CCR5 utilizing M tropic strains that are seen during early stages of infection. The strains that use CXCR4 are known to exhibit cytopathic effect *in vitro* [19]. However, this observation might be an *in vitro* artifact since little cytopathic effects were noticed *in vivo *[20]. There is differential usage of CXCR4 coreceptor among subtype C and D viruses, with subtype C viruses rarely switching to CXCR4 usage and subtype D viruses utilizing CXCR4 receptor earlier and frequently, and this, alongwith other factors like genetic variation in long terminal repeat (LTR) promoter, might account for their varied impact on disease progression [8, 21–24]. The promoter/enhancer activities of LTRs of subtype C viruses were shown to be higher than that of other subtypes A, B, D, E, and G [23], and subtle differences in promoter activity of LTRs might affect HIV replication kinetics substantially [24, 25]. The intrasubtype diversity is substantial among different subtypes of HIV-1. The protein sequence diversity among subtypes of HIV-1 group M viruses for Gag, Pol, and Env are reported to be 15%, 10%, and 24%, respectively [26]. Gag-30 position is phylogenetically important. The sequences of SIV_CPZ_ *Ptt*, from which HIV-1 originated, are marked by presence of Met at Gag-30. In contrast, the ancestors of all the three HIV-1 groups (M, O, and N) are marked by sequences that contain Arg at Gag-30, highlighting potential host-species-specific adaptation [27]. Understanding HIV evolution and the role that the host plays in mediating it and controlling infection will undoubtedly help to determine the effective approaches to combat HIV/AIDS.

## 3. Degree of HIV-1 Variability

**Figure 3:**
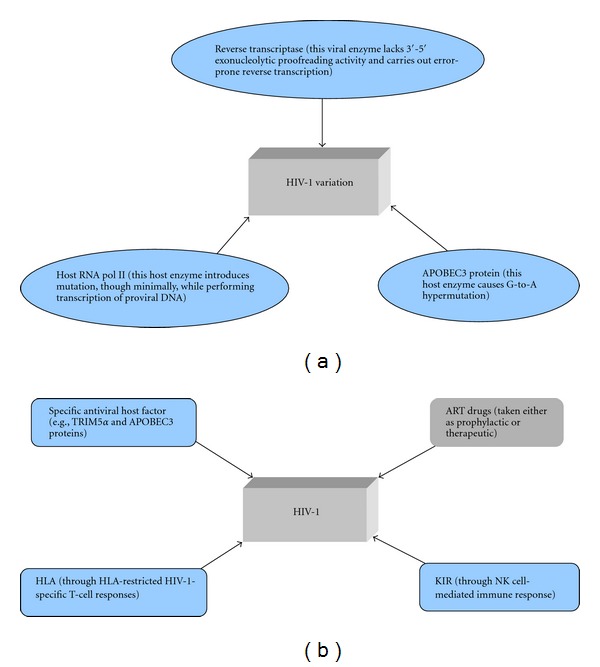
(a) Error-causing machinery involved in HIV-1 mutagenesis. (b) A schematic sketch of selection pressures acting on HIV-1. Note: ART drugs block is shown in grey colour to differentiate from others, as such drugs exert influence over HIV-1 indirectly in patients undergoing ART.

HIV-1, with its RNA genome, demonstrates significant genetic diversity due its high mutation rate. It has diversified itself to such an extent, through its ability to form “cloud” of variants or quasispecies, that there is no single wild-type strain. *In vitro *data have shown that RNA viruses generate nonhomogeneous genetic clones that are closely related but genetically diverse, which are known as quasispecies. This phenomenon, which aids viruses to persist in their host, possibly causing disease, is observed in other RNA viruses such as hepatitis C and influenza virus as well [28, 29]. The reverse transcriptase (RT) of HIV-1, which lacks 3′-5′ exonucleolytic proof-reading function, misincorporates 1 in 6900 and 1 in 5900 nucleotides polymerized on the RNA and DNA template, respectively, and hence accounts for larger proportion of mutations seen in HIV-1 [30]. It has been estimated that, after a single round of HIV-1 replication, under the assumption of absence of selection pressure, the resulting progeny viruses will have substitution, frameshift and deletions at 24%, 4%, and 2%, respectively [31]. It is interesting to note that 80% of heterosexual-mediated HIV-1 infections are due to productive infection by a single HIV-1 virion [32–34]. HIV-1 evolves at about 1% per year [35]. Given that HIV-1 faces selection pressures, a gamut of mutations has shaped its genome since its origin, which in turn, ensures its virulence at population level [36, 37], despite the fact that certain mutations in its conserved region impacts its fitness negatively [38]. Further, a recent study that utilized phylogenetic comparative approach revealed that viral genotype, as against the host genetic profile, largely determines the HIV set-point viral load and hence the virulence [39]. A schematic sketch of error-causing machinery involved in HIV-1 mutagenesis and a gamut of selection pressure acting on HIV-1 are provided in Figures 3(a) and 3(b), respectively.

### 3.1. Ultra-Deep Sequencing Reveals Vastness of HIV-1 Variability

With the emergence of new pyrosequencing technology, HIV-1 viral quasispecies are now more rapidly and accurately sequenced and analyzed. Mutation spectra of HIV-1 quasispecies are wide, and traditional sequencing methodologies are limited in their ability to capture minority variants [40]. Next generation sequencing (NGS) methodologies have made it possible to obtain high-throughput sequence data at an unprecedented pace and coverage (e.g., pyrosequencing using GS FLX+ system permit characterizing up to 1000 bp read length with 1,000,000 reads per run at run time of 23 hours and consensus accuracy of 99.997%) [41, 42] and are being employed to decipher HIV-1 evolutionary trajectories [34, 43]. Recently, Liang et al. [44] used 454 pyrosequencing technology and sanger clone-based sequencing to assess the genetic diversity of HIV-1 gag and it was determined that pyrosequencing detected almost four times more variation in gag than sanger sequencing. Ultra-deep sequence sets of HIV-1 allow for deciphering CTL escape variants that are not discernable with the sequences obtained through conventional sequencing strategies [34, 44]. While single genome amplication (SGA) is superior to standard genotyping method [45], ultra-deep sequencing methods offer highest sensitivity to date in relation to those conventional methods as it can detect minor viral variants that comprise lower than 1% of the population. This highest level of sensitivity by ultra-deep sequencing also allows for identifying low-abundance drug resistant variants [40], with potential to interfere with ART outcome. Not only NGS techniques are used to gain insights into the sequence of the viral genome with greater depth, it has also been recently utilized to examine viral diversity after therapy. For example, a study that used deep sequencing technology to examine escape mutations in the V3 loop of HIV-1 that arise as a result of selection by CCR5 antagonist (vicriviroc, a drug that inhibits HIV-1 entry) therapy indicated significantly higher sequence heterogeneity [43]. Knowledge on nature of HIV-1 quasispecies gained through ultra-deep sequencing technologies can aid in progressing HIV research and managing HIV/AIDS clinically better. Finally, with advanced whole genome sequencing technologies, the ability to correlate genome profile of HIV with that of patients could lead to comprehensive understanding of disease process and effective interventions.

### 3.2. Factors Driving HIV-1 Variability

**(a) Inherent Property of the Reverse Transcriptase (RT) and Recombination**

The generation of diverse variants in HIV-1 can be attributed mainly to its low-fidelity RT enzyme, leading to error-prone reverse transcription [30]. RT also accounts for genomic heterogeneity in progeny viruses through its role in recombination. Besides RT, which accounts for larger proportion of mutations observed in HIV-1, the host RNA pol II involved in transcription of proviral DNA can also contribute to mutations, albeit minimally. A study has indicated contribution of cellular RNA pol II to be less than 3% of retroviral frame-shift mutations [46].

HIV-1, with documented dual and triple infections in patients [47, 48], can substantially drive production of viral quasispecies that are endowed with superior fitness through the process of genetic recombination, a time-tested evolutionary strategy to thrive in a changing environment. With an average of 1.38 × 10^−4^ recombination events/adjacent sites/generation *in vivo* [49], HIV-1 ensures its ability to enrich both diversity and fitness. In HIV-1, recombination in genomic regions with high selection pressure, either in the form of host immune response or ART drugs, could lead to selection of more fit genomes, while, in regions under negligible selection, recombination can enhance diversity [50]. HIV-1-infected commercial sex workers in Nairobi, Kenya, were shown to harbor high proportion of recombinant HIV-1 viruses [51, 52]. Recombinants between highly similar HIV-1 strains are formed at highest frequencies while that between very distant HIV-1 strains occur at very low frequencies [53]. Genetic recombination between HIV-1 and HIV-2 is also a potential possibility [54].

**(b) Swift Turnover Rates of HIV-1 In Vivo**

HIV-1 virions are produced and cleared at extremely rapid pace. HIV-1 turnover is high at 10^11^ virions and 10^8^ infected cells per day [45]. Studies have estimated that free HIV-1 viral particles have an half-life of less than 6 hours, while the productively infected cells possess an half-life of about 1 day [55]. This rapid turnover has been considered as the major factor underlying pathogenesis of HIV/AIDS, wherein there is greater destruction of CD4^+^ T helper lymphocytes.

**(c) Drugs of ART Drive Changes in HIV Genetic Makeup**

Antiretroviral drugs as well as associated drug resistance mutations could influence *in vivo *HIV-1 mutation rates. The drug 3′-azido-3′-deoxythymidine (AZT) can enhance HIV-1 mutation rate by a factor of seven per round of replication, and HIV-1 variants harbouring AZT resistant RT can incur higher mutation rate as much as threefold relative to wild-type RT bearing HIV-1 [56]. The V106A is a nevirapine (nonnucleoside RT inhibitor) resistance mutation that has been consistently shown to affect viral fitness severely [57]. Mutations associated with HIV-1 drug resistance could be present in drug-naïve patients at low frequencies, often interfering with outcome of ART [58]. Metzner et al. [59] have reported occurrence of M184V (RT) and L90M (protease) mutations as minority populations in patients undergoing structured treatment interruptions. A comprehensive list of ART drug-associated mutations is being maintained in Stanford HIV Drug Resistance Database [60]. Further discussion on ART drugs is covered in Section 6.

**(d) Selective Immune Pressures from Host**

There is heterogeneity in disease progression among HIV-1-infected patients. Host genetic variants have been shown to account for at least 15% of the observed differences in disease progression [61]. Human leukocyte antigen (HLA) system, residing in the sixth chromosome, is the most polymorphic loci in human genome and this extensive polymorphism is the result of evolving with millions of pathogens that the human species has faced throughout its existence [62, 63]. This elaborate polymorphic nature of HLA alleles enables them to recognize various protein fragments of pathogens and present to T cells for generation of appropriate immune responses. Among these host genetic factors, HLA class I molecules, which present peptides to cytotoxic T cells, have been shown to exert profound influence over controlling the HIV-1. The protective alleles, HLA-B*57, -B*27, and -B*51, through presentation of highly conserved HIV-1 epitopes to cytotoxic T lymphocytes (CTL) and subsequent immunodominant immune response, drive formation of specific CTL escape mutants, that are compromised in their replicative fitness [64–68]. These CTL escape mutants, depending on nature of HLA-mediated immune pressure from subsequent hosts in the transmission chain, may get either fixed in the population or get reverted to their wild type form [38, 69]. Selection pressure exerted by host on HIV-1 is discussed further under the subsequent Section 4.

## 4. Host Genetic Factors Influence HIV-1 Evolution

HIV-1 adapts to host immune pressure, and this is revealed through studies of positively selected amino acid changes in different proteins of the virus [70–74]. An immunoinformatic analysis that looked at envelope sequences across clades from varied geographical regions has indicated differences in frequency of positive selection (PS) sites, suggesting that viral clades prevalent in various geographically distinct parts of the globe evolve in response to the characteristic immunogenetic profile of the host population [70]. Evolutionary pathways of HIV-1 appear to be vast with occurrence of positive selection sites not only in epitopes of CD4^+^ and CD8^+^ T cells and antibodies, wherein HLA impacts profoundly, but also in other regions which are likely to suffer selection pressure via effectors of innate arm of the immune system such as KIR and HIV restriction factors TRIM5*α*, APOBEC3G [75]. In contrast, HIV-2 that causes less severe form of disease face significant negative selection pressure [76]. 

Several AIDS restriction genes have been identified [77, 78]. Among these HLA, killer-cell immunoglobulin-like receptors (KIRs), chemokine receptors and intrinsic antiviral factors like TRIM5*α*, APOBEC3 are known to exert substantial influence over HIV/AIDS and affect HIV-1 evolution. TRIM5*α*, for example, has the ability to recognize the capsid protein of the incoming virus and disassembling it upon entry [79]. APOBEC3 proteins are another group of host restriction factors that play a role in reducing viral infection, including HIV-1 [80]. These host proteins are cytidine deaminases that catalyze the deamination of cytidine to uridine, which results in guanosine to adenosine hypermutation in the concerned opposite strand, favouring inactivation of the virus [79, 81]. HIV-1 negates the antiviral effects of APOBEC3G (A3G) through its Vif (viral infectivity factor). Viral infectivity factor promotes proteosomal degradation of A3G in an incomplete fashion, as a result of which there is generation of hypermutated viral population that could still survive, aiding HIV-1 evolution and possibly favouring emergence of drug resistant forms [82]. Fourati et al. [83] showed that HIV patients exhibiting virological treatment failure often possessed K22H point mutation in Vif, which resulted in inability of Vif to counteract APOBEC3 proteins, ultimately leading to G-to-A hypermutation in HIV. Several other studies have also demonstrated role of APOBEC3 proteins in HIV-1 evolution [84–87]. Recently, study by Norman et al. [88] indicated that HIV-1 also employs viral protein R (Vpr) to negate A3G antiviral properties by diminishing the incorporation of uridines in the deamination process. Interestingly, this act by Vpr results in favour of host as DNA damage response pathway got triggered and NK cell-activating ligands got expressed, making the virus vulnerable to attack by NK cells [88]. Human tetherin also acts to prevent the spread of HIV-1 infection to other cells. Tetherin, a cell surface host protein, is able to trap virions that are being released from the surface of the infected cell [79, 89]. Differential adaptation of HIV-1 viruses to antiviral activity of tetherin has been recently confirmed. Yang et al. [90] demonstrated that while Vpu of both group M and N HIV-1 viruses had activity against human tetherin, Vpu and Nef from group O and P viruses lacked such anti-tetherin activity. A very recent study by Liu et al. [91] reported identification of 114 intrinsic host factors with significant ability to inhibit HIV-1 infection and this illustrates the tremendous pressure HIV-1 is subjected to, upon entry into its host. It could be inferred that various host genetic factors might additively contribute to controlling the virus. The complex HIV-1 host interactions are being dissected using genome-wide and large-scale strategies to map virus-host interactions comprehensively [92].

### 4.1. HLA Leaves Footprints on HIV-1

The impact of HLA diversity on HIV evolution has been documented in several studies. Different HLA alleles have been shown to be associated with different rates of HIV disease progression. For example, patients who possess HLA-B*27 and -B*57 alleles normally have low viral loads and progress to AIDS at a much slower rate, while those possessing HLA-B*35 progress to AIDS defining illnesses rapidly [64]. HIV-1 is under pressure from HLA-mediated CTL responses quite early in the infection as CTL escape mutations have been shown to arise as early as 14 days of postinfection [93]. CTL epitopes, are reported to be more conserved compared to CD4^+^ T helper and monoclonal antibody epitopes and this conservation of CTL epitopes has been suggested as a host strategy to constrain HIV-1 adaptation [94]. Evidence of HLA footprint on HIV-1 genome is demonstrated by studies that have investigated the mutation profile of original infecting HIV-1 strains. Leslie et al. [95] analyzed the mutation of clade B and C HIV-1 in patients with HLA-B*57/58 : 01 allele, which are associated with slow progression to AIDS. It was observed that positively selected amino acids had accumulated and, once transmitted to HLA-B*57/B*58 : 01 negative individuals, the virus reverted back to its wild-type form [95]. This illustrates the ability of HLA alleles to drive the necessary mutation in HIV, as part of controlling the infection. In another study, the carriage of HLA-B*57 allele in patients infected with HIV-1 and its impact on viral control was assessed. It was demonstrated that individuals expressing the HLA-B*57 allele controlled viremia without therapy at levels <5000 copies/mL of virus for upto 29 months, and a stronger and broader response was generated by HLA-B*57 allele than other HLA class I alleles [96]. A Swiss HIV cohort study, that reported similar transcriptome profile of CD4^+^ and CD8^+^ T cells among rapid progressors and pathogenic SIV-infected rhesus macaques, also found underrepresentation of protective alleles and overrepresentation of risk alleles at HLA loci in rapid progressors [97]. HLA selection pressure on HIV-1 is so fine-tuned that micropolymorphism seen among subtypes of a particular allele could exert differential pressure on virus. This phenomenon has been recently demonstrated for the HLA-B*57 alleles [98]. The support for extensive HLA associated selection in HIV-1 is also evident in the recent study by Dong et al. [99], wherein they followed a narrow-source HIV-1 outbreak, that occurred through a plasma donation scheme in a Chinese village and found 24–56% of the polymorphic sites across Gag, reverse transcriptase, integrase, and Nef had HLA footprints. A comprehensive genomewide association analysis has revealed that amino acids at positions 67, 70, and 97 in HLA-B play a major role in determining HIV-1 control, given their involvement in peptide binding within the peptide binding groove [100]. 

HLA-B loci, the most rapidly evolving class I region, contains alleles that exert strong selection pressure over HIV-1 through their allele-restricted CD8^+^ T cell responses and contributes to shaping of HIV-1 evolution [101, 102]. HLA-B might predominantly shape HIV and vice versa, a coevolution scenario [67, 101] as exemplified by Red Queen Hypothesis [103]. Also rapid selection for HLA alleles that protect against HIV-1 infection has been found to correlate significantly with declining incidence of HIV-1 in an East African sex worker cohort of Kenya, which suggests that natural selection might eventually play a vital role in containing the HIV-1 epidemic [104]. It may be plausible that mutome of HIV and human is being shaped by each other in a very delicate dynamic process of virus-human partnership.

Intrinsic and adaptive immunity might work synergistically to contain HIV-1. This can be inferred from the studies that have dissected role of HLA and KIR compound genotypes over HIV disease progression [105]. Further, recently CTL escape mutations in Gag have been shown to enhance sensitivity of HIV-1 to TRIM5*α* [106]. It is plausible that HIV-1 could suffer a double whammy attack-one, fitness cost due to mutation in a highly conserved region and, second, increased vulnerability to attack from TRIM5*α*. In order to survive host immune pressure, which is predominantly dictated by HLA, HIV mutates at specific epitopic region and this escape variant to survive further without compromising its fitness might undergo compensatory mutations in regions away from the concerned epitope. Though HIV-1 mutates rapidly as a stochastic process, its mutational strategies are relatively predictable. The studies employing HIV-1-infected identical twins suggested the presence of a relatively narrow window period in HIV infection, wherein the immune responses, viral evolution as well as disease progression are somewhat reproducible and hence predictable [107–109]. Moreover, recently Dahirel et al. [110] have elegantly carried out coordinate linkage analysis employing a physics concept to highlight multidimensionally constrained regions of HIV-1 proteome. They have identified HIV sectors that is, distinct sets of amino acids whose mutations are collectively coordinated and indicated that among the five sectors of Gag, sector 3, which plays vital role in assemblage of multiprotein structures for formation of HIV-1 capsid, is the most immunologically vulnerable multidimensionally constrained and also is the sector most targeted by elite controllers of HIV-1, who harbour protective HLA alleles. These studies bear potential clues for designing successful anti-HIV immunogens. While role of HLA class I alleles in attenuating HIV-1 is vastly studied and supported by several findings, part played by HLA class II alleles has been scarcely investigated [64, 111, 112]. A recent study that investigated the correlation between HLA class II alleles and *in vitro* replication capacities of recombinant viruses encoding Gag-protease from HIV-1 subtype C infected chronic patients failed to detect any association of alleles with lower fitness [113]. However, earlier studies have demonstrated potential role of HLA class II alleles in exerting selection pressure on HIV-1 [114, 115]. More studies are warranted, given the reported significant genetic associations of alleles belonging to HLA-DR,   -DQ, and -DP loci with HIV infection and disease [64, 111, 116–120], to delineate degree of immune pressure exerted by different HLA class II alleles, the players in generating the essential T helper cell responses. T-cell-based vaccine strategies that could address HIV-1 diversity issues better are being tested [121, 122].

### 4.2. KIR Footprints on HIV-1

Killer-cell immunoglobulin-like receptor (KIR) encoding genes are located on chromosome 19, and their major role is to control the activation or inhibition of Natural Killer (NK) cells, which belong to the innate arm of the immune system. KIRs are quite polymorphic, and thus they are able to generate a diverse response to a variety of pathogens. KIRs mediate their effects using HLA molecules as ligands [123, 124].

HIV-1, like other viruses, down-regulate HLA class I molecules, specifically HLA-A and -B, and hence escapes from those HLA-mediated CTL effectors. However, in order to escape attack by NK cells, which destroys target cells lacking expression of HLA class I molecules, HIV-1 avoids downregulating KIR-interacting HLA-C or the nonclassical HLA-E molecules [125]. KIRs are known to impact HIV-1 disease outcome both independently and synergistically through its interaction with HLA ligands [126, 127]. A recent study has demonstrated role of copy number variation in KIR genes in influencing HIV-1 control [128]. Alter et al. [129] have shown that HIV-1 evades NK cell-mediated immune response by selecting for viral variants that modulate recognition of infected cells by KIR to their advantage. Specifically they identified 22 KIR-associated polymorphisms in HIV-1 from a cohort of 91 untreated chronically HIV-1-infected patients. HIV-1 viruses with Vpu (71 M/74 H) (Env (17 W/20 M)), Gag (138I), and Nef (9 K) were found to be significantly enriched in individuals possessing KIR2DL2, and these KIR footprints enhanced the binding of inhibitory KIR to infected cells, due to which inhibition of NK cell function ensues and HIV-1 escapes attack [129].

## 5. Problems Posed by HIV-1 Diversity

### 5.1. Search for a Broadly Cross-Reactive Anti-HIV Neutralizing Antibody

HIV-1 diversity is one among several challenges that needs to be addressed while attempting to design an effective anti-HIV vaccine. Generating broadly neutralizing antibodies (bnAbs) that can effectively inactivate or neutralize HIV variants remains elusive [130]. Broadly neutralizing antibodies are rare and undetectable in most HIV-1-infected individuals. Several hypotheses exist that attempt to explain the rarity of bnAbs. For example, one reason that has been proposed is that highly immunogenic epitopes may trigger nonneutralizing antibodies instead of activating required specific responses [131, 132]. However, nonneutralizing antibodies could be functional against HIV-1, as observed in study subjects of RV144 Trial [133], and have potential to mediate protection against HIV-1. Studies have also shown that antibodies sometimes select for escape mutations [134, 135]. There are four regions of HIV-1 Env that could serve as targets for bnAbs: gp120 CD40 binding site, quaternary V2/V3 loop epitopes, gp41 Membrane proximal external region (MPER), and Env carbohydrates [132]. Human immunoglobulin, VRC01, is capable of neutralizing 90% of the HIV-1 isolates [136]. Recent studies have delineated both evolutionary course and nature of VRC01-like antibodies [137, 138], and this knowledge has opened up new avenues for strategies to attack HIV-1 better.

### 5.2. Correlates of Protection Obscured by HIV-1 Evolution

Certain host genotypes known to be favourable prior to ART might turn out to exert a detrimental or neutral effect upon initiation of treatment. This intriguing observation is being reported frequently, and yet the mechanism underlying the association is unclear. Rauch et al. [139] reported that Bw4 homozygosity, associated with protection in untreated patients, predicted impaired CD4 T-cell recovery upon commencement of combination ART. Another study revealed strong association of HLA-B*57 : 01 and -B*58 : 01, both exhibiting Bw4 motifs, with failure to control HIV replication following HAART initiation [140]. While HAART exhibits the potential to suppress HIV replication profoundly irrespective of the genotype of the individual, documented association of specific highly protective alleles with differential outcome over treatment may have an unidentified functional immunological basis and warrants extensive investigation. Antiretroviral therapy-induced selection pressure on *pol* could lead to generation of HIV-1 quasispecies with significant changes in epitope profile, including loss of protective epitopes. Furthermore, HLA-KIR interaction might contribute to outcome of ART [139, 140] and may explain the conundrum of what is good before ART is not so after ART.

HLA-B*51 has been associated with protection against HIV-1 in Asian population [67, 68]. This allele has been able to confer a multilayered defence against HIV/AIDS through presentation of highly conserved immunodominant epitopes in Gag region, that rarely undergo mutation, and, if at all gets mutated, it is only at the cost of fitness. However, it has been noted that, over a period of time, at population level, the circulating viruses, as they evolve, tend to lose the epitopes targeted by the protective alleles such as HLA-B*51 [67, 68, 141], such that documented protective association is obscured and these evolutionary strategies by which HIV changes its genomic/proteomic landscape to stay ahead, pose tremendous challenge for scientists as they search for true correlates of protection against HIV/AIDS and venture into developing a stable and effective intervention-prophylactic/therapeutic vaccine [142, 143]. Interestingly, a mathematical modeling study has predicted that the rate of generation of escape mutants and the transmission of escape mutants may have only a weak impact on the epidemic outcome over the first 25 years after the introduction of a nonsterilizing anti-HIV vaccine [144]. However, search for a sterilizing vaccine for HIV/AIDS, a holy grail, remains vital aim in fight against HIV [145]. 

Characterizing immunological profile in both elite controllers and HIV-exposed seronegatives could lead to better understanding of correlates of protection [143, 146–151]. Additionally, understanding immunobiological basis of benign nature of disease induced by HIV-2 can provide clues into virus-host interaction and aid in tackling HIV-1. Expanding HIV-1 diversity might pose problems at diagnostic level given its impact on viral load testing assays [152].

## 6. Scope of ART in HIV-1 Control

According to UNAIDS world AIDS day report 2011, at least 6.6 million people in low- and middle-income countries are receiving HIV treatment and this has resulted in prevention of 2.5 million AIDS deaths since 1995 [2]. Also ART prevents infection, as it reduces viral load and infectiousness of an infected individual [153]. While this is an encouraging sign towards combating the HIV/AIDS epidemic, it is to be emphasized that current drugs in the prescribed regimen are unable to attack and eradicate the viruses hiding in reservoirs such as seminal vesicles [154] and tissue macrophages of HIV infected patients [155]. Given the evidences that suggest continual on-going replication of HIV-1 in such reservoirs [156, 157], it is plausible that quasispecies that are immune to current combination ART drugs can emerge upon treatment interruption. HIV-1 occupies variety of anatomic compartments such as central nervous system (CNS), gut-associated lymphoid tissue (GALT), and genitourinary tract [158, 159]. The CNS, endowed with blood-brain-barrier, is a pharmacologically “privileged” site, and the virus inside CNS thus gets shielded from attack by some ART drugs [159–161]. Genotypic diversity of HIV-1 is not uniform across different compartments [162]. This can be inferred by the fact that majority variant seen in blood is not always so in semen [163, 164]. Further, *env* sequences from blood and male genital tract compartments differ [165]. Venturi et al. [166] have observed different drug resistance mutation profile between HIV-1 isolates from cerebrospinal fluid and plasma in patients under nonsuppressive ART drug regimens. Indeed selective drug pressure has been shown to result in multiple drug-resistant HIV-1 quasispecies [167]. Viral rebound in patients who cease to continue with the ART is an added concern [168]. 

A recent study that evaluated the correlation of preexisting drug-resistant HIV-1 minority variants with risk of first-line nonnucleoside reverse transcriptase inhibitor (NNRTI) based antiretroviral virologic failure, by reviewing 10 different studies, has suggested significant association of low-frequency drug resistance mutations with a dose-dependent increased risk of failure to control the virus [169]. Another cause for concern is the differential persistence of transmitted HIV-1 drug resistance mutation classes as reported by Jain et al. [170], wherein they indicated long-term persistence of NNRTI and protease inhibitor mutations which might facilitate person-to-person propagation. Adherence to drug regimen among HIV-1 patients is threatened by the fact that certain prescribed combination ART drugs could induce unfavourable side effects among patients with specific genotypes. For instance, hypersensitivity reactions (HSRs) are seen in HLA-B*57 : 01 positive HIV-1 patients receiving Abacavir (ABC), an nucleoside reverse transcriptase inhibitor (NRTI) drug [171]. While there are similar effects documented for other ART drugs [172], the association of ABC with HSR in HLA-B*57 : 01 patients is robust enough that screening for this allele has been made routine prior to prescription of combination ART regimen containing ABC [173]. More insights into pathophysiology of drug-induced HSR in HIV patients can ensure case-specific recommendation of combination ART drugs, averting compliance issues and emergence of drug resistant viral population. He et al. [174], by conducting a 7-year follow-up study on 437 HIV-infected Chinese patients undergoing HAART, suggested that two NRTIs and one NNRTI regimens could persistently suppress HIV viremia and enhance CD4^+^ T-cell population with good safety and tolerance. The study also reported 19.2% of the participants changed to other first-line drug due to drug-related side effects and 10.2% switched to second-line regimens because of viral resistance. As UNAIDS and WHO advocate Treatment 2.0 [175], many such prospective studies analyzing outcome of HAART regimens are essential and antiretroviral pharmacovigilance [176] will attain greater importance. 

HIV drug resistance could be either acquired or transmitted. According to recent initial survey conducted in low- and middle-income countries, WHO has reported acquired HIV drug resistance rate to be 6% while 3.7% rate for the transmitted HIV drug resistance [177]. The 2009 Surveillance Drug Resistance Mutation (SDRM) list has indicated 93 mutations including 34 NRTI-resistance mutations (at 15 RT positions), 19 NNRTI-resistance mutations (at 10 RT positions), and 40 PI-resistance mutations (at 18 protease positions), and this suggests the vast number of mutations linked to antiretroviral drug resistance [178]. The fitness landscape of HIV-1 RT and protease has been shown to be under strong epistasis [179]. Epistasis refers to a situation wherein action of one genetic locus masks the allelic effects at another locus, and the locus thus masked is referred to be “hypostatic” to the other locus [180, 181]. This phenomenon of epistatic interaction complicates comprehensive understanding of viral variants and their relationship to drug resistance. Even though today there are more than 20 different antiretroviral drugs to treat HIV-infected patients, a major global public health concern is the emergence of new strains that develop resistance to these drugs and subsequent transmission to other hosts [176]. 

Antiretroviral therapy has been quite successful which can be attributed to its ability to control HIV replication and preserve optimal CD4^+^ T helper cell population, due to which many of the opportunistic infections associated with abnormal low CD4^+^ T-cell counts are averted. However, tuberculosis (TB), caused by *Mycobacterium tuberculosis*, can occur at any stage of the disease, irrespective of CD4^+^ T-cell counts, in HIV-1-infected patients [182] and poses a tremendous public health challenge in regions plagued by dual epidemic of HIV and TB. HIV-associated TB and hepatitis complicate the clinical management of individuals suffering from such coinfections, with potential for development of immune reconstitution inflammatory syndrome and drug-drug interactions are not clearly understood [183, 184]. 

HIV-1 latency presents challenges for the attempts directed at eradicating it. The half-life of the latent, replication-competent HIV-1 in resting CD4^+^ T cells is roughly six months, which necessitates compliance to effective ART regimen for several years to clear the virus from reservoir [157]. Strategies are being contemplated to activate the HIV residing in latent reservoirs, in such a manner that does not allow wide-spread infection of uninfected cells [185, 186], and, in this regard, current and future ART drugs could play vital role in eradicating resilient virus.

## 7. Conclusion

The studies on biology of HIV-1 variation by characterizing emerging quasispecies population carries prognostic value as they impact rate of development of AIDS defining illnesses [187] as well as effectiveness of therapy [188]. Different recombinant forms of HIV-1 emerge, and they are seen to predominate in particular environments [189–191]. Given this scenario, more comprehensive epitope mapping studies with focus on CTL epitope escape mutants of CRFs, in addition to characterizing epitope profile of major HIV-1 clades, are warranted, and such findings will augment the efforts to curb spread of HIV-1, a virus nonpareil in medical history due to its ever elusive tricks inflicting damage to global health. The advent of HAART has made HIV/AIDS a life-threatening fatal into a potential chronic disease. However, HIV patients under long-term treatment are likely to have higher risk for medical complications like heart, liver, and neurodegenerative diseases, and hence there is an increasing need to deal with these additional health issues effectively [192]. A vaccine that could elicit sterilizing immunity against HIV/AIDS is much desired. Recent findings, RV144 Trial, with its finding that prime-boost vaccine combination of ALVAC-HIV and AIDSVAX^®^ B/E offering 39.2% protection against HIV [193]; a 1% tenofovir gel inhibiting HIV sexual transmission by 39% [194]; a person with 12 years of infection considered to be cured of HIV as part of fighting acute myeloid leukemia via haematopoietic stem cell transplantation from a CCR5 ∆32 homozygous donor [195–197] are quite encouraging and serve as testimonial to the fact that HIV-1 can be conquered through further research which includes dissecting mechanisms of underlying protection and moving forward with those anti-HIV immunobiological clues [198–201]. While debate on attenuation of HIV-1, as it evolves continues [36, 37, 202, 203], focused and concerted efforts by scientists, employing multidisciplinary approaches to attack HIV, might enable achieving UNAIDS mission of “zero new HIV infections, zero discrimination and zero AIDS-related deaths” earlier.
